# Epidemiology and molecular detection of human adenovirus and non-polio enterovirus in fecal samples of children with acute gastroenteritis: A five-year surveillance in northern Brazil

**DOI:** 10.1371/journal.pone.0296568

**Published:** 2024-08-02

**Authors:** Jainara Cristina dos Santos Alves, Dielle Monteiro Teixeira, Jones Anderson Monteiro Siqueira, Danielle Rodrigues de Deus, Darleise de Souza Oliveira, James Lima Ferreira, Patricia dos Santos Lobo, Luana da Silva Soares, Fernando Neto Tavares, Yvone Benchimol Gabbay

**Affiliations:** 1 Postgraduate Program in Virology, Evandro Chagas Institute, Secretariat of Health and Environmental Surveillance, Ananindeua, Pará, Brazil; 2 Virology Section, Evandro Chagas Institute, Secretariat for Health Surveillance and Environment, Ananindeua, Pará, Brazil; PearResearch / Government Doon Medical College, INDIA

## Abstract

Acute gastroenteritis (AGE) is a common pediatric infection that remains a significant cause of childhood morbidity and mortality worldwide, especially in low-income regions. Thus, the objective of this study was to detect human adenovirus (HAdV) and non-polio enterovirus (NPEV) in fecal samples from the Gastroenteritis Surveillance Network, and to identify circulating strains by nucleotide sequencing. A total of 801 fecal samples were tested using qPCR/RT-qPCR, and 657 (82.0%) were inoculated into HEp-2C and RD cell lines. The HAdV and NPEV positivity rates obtained using qPCR/RT-qPCR were 31.7% (254/801) and 10.5% (84/801), respectively, with 5.4% (43/801) co-detection. Cytopathic effect was observed in 9.6% (63/657) of patients, 2.7% (18/657) associated with HAdV, and 6.2% (41/657) associated with NPEV after testing by ICC-PCR. A comparison of the two methodologies demonstrated an agreement of 93.5% for EVNP and 64.4% for HAdV. These two viruses were detected throughout the study period, with HAdV positivity rates ranging from 41% in Amapá to 18% in Pará. The NEPV varied from 18% in Pará/Rondônia to 3% in Acre. The most affected age group was over 60 months for both HAdV and NPEV. Samples previously positive for rotavirus and norovirus, which did not show a major difference in the presence or absence of diarrhea, fever, and vomiting, were excluded from the clinical analyses of these two viruses. These viruses circulated over five years, with a few months of absence, mainly during the months corresponding to the waves of SARS-CoV-2 infection in Brazil. Five HAdV species were identified (A, B, C, D, and F), with a greater predominance of HAdV-F41 (56.5%) followed by HAdV-C (15.2%). Three NPEV species (A, B, and C) were detected, with serotypes E14 (19.3%) and CVA-24 (16.1%) being the most prevalent. The present study revealed a high diversity of NPEV and HAdV types circulating in children with AGE symptoms in the northern region of Brazil.

## Introduction

Acute gastroenteritis (AGE) is a common pediatric infection that remains a significant cause of childhood morbidity and mortality worldwide, affecting children under five years of age, especially in low-income regions [1]. It is characterized by a rapid-onset diarrheal disease with or without nausea, vomiting, fever, or abdominal pain, with an increase in the number of bowel movements and changes in the consistency of feces without association with any previous chronic disease [2]. AGE can be caused by a wide spectrum of pathogens, with enteric viruses responsible for many diseases [3].

Among enteric viruses, group A rotavirus (RVA) is considered the main cause of infection in children younger than five years, followed by norovirus (NoV), astrovirus, and human adenovirus (HAdV) [4–7]. However, other viruses, including sapovirus and enterovirus (EV), have been associated with AGE. These viruses are mainly transmitted via the oral fecal route [8, 9].

HAdV belongs to the family *Adenoviridae* and genus *Mastadenovirus* and is a non-enveloped icosahedral virus containing a linear double-stranded DNA genome of 34–36 kb in size and a diameter of 70–90 nm. It is currently divided into seven species termed A-G including more than 100 different types [10–12].

Clinical diseases and epidemiological characteristics of HAdV-related infections report the presence of conjunctivitis outbreaks associated with aquatic recreational activities or military areas (HAdV-B and HAdV-E), as well as acute (HAdV-B) or endemic respiratory diseases (HAdV-C) in children, in addition to outbreaks of keratoconjunctivitis. Gastroenteritis is common in patients with AIDS (HAdV-D) and in children, especially in those caused by species F [12]. HAdV F40 and F41 is frequently detected in cases of acute gastroenteritis in young children and are one of the main causes of childhood viral diarrhea (children under two years old), whereas species A and E are associated with rare cases of gastroenteritis [10, 12].

EV belong to the family *Picornaviridae* and genus *Enterovirus*, which are single-stranded, small, non-enveloped enteric RNA viruses that usually cause infections in children. Human EV are classified into four species (EV-A to -D) and include more than 100 types [13]. Poliovirus belongs to HEV-C and is the best-known neurovirulent EV, with endemic poliomyelitis being eradicated in almost all countries [14]. Although most infections are asymptomatic, the clinical manifestations of EV infections can range from mild (respiratory and gastrointestinal infections, hand-foot-and-mouth disease, and herpangina) to severe (pleurodynia, hepatitis, myopericarditis, pancreatitis, meningitis, encephalitis, paralysis, and sepsis). Currently, worldwide EV surveillance networks are available to prevent and control outbreaks of EV-related diseases [15].

Non-polio enteroviruses (NPEV) such as EV-D68, EV-A71, and some coxsackieviruses have been associated with acute flaccid myelitis, a disabling polio-like illness that frequently affects young children and is characterized by acute onset flaccid weakness of one or more limbs [16]. Other NPEV, such as echovirus, are commonly associated with gastroenteritis, meningitis, and respiratory illness, and have most recently been associated with neonatal cases of severe hepatitis [15, 17].

In recent years, studies on the molecular epidemiology of HAdV in cases of gastroenteritis have been performed more frequently in the southern and southeastern regions of Brazil, before and after the implementation of the rotavirus vaccine [18, 19] with few reports in the northern region [20]. NPEV has already been detected in fecal samples from the northern region of Brazil; however, its relationship with gastroenteritis cases is not well understood [21, 22].

To provide more information about HAdV and EV circulation in the northern region of Brazil, fecal samples from children attended by the Gastroenteritis Surveillance Network from 2017 to 2021 were evaluated by molecular methods for viral detection, and the types were identified by partial nucleotide sequencing. Furthermore, this study also aimed to associate sociodemographic variables, such as age and sex, clinical aspects, and previous rotavirus vaccination status, with positive cases for HAdV and EV.

## Methods

### Ethical aspects and biosafety

This study was approved by the Ethics Committee of the Evandro Chagas Institute (IEC), Brazil (approval number: CAAE 94144918.3.0000.5248) and was conducted in accordance with its regulations. Fecal samples were handled anonymously, and patient data were kept secure. The IEC Ethics Committee waived the requirement for informed consent. Personal protective equipment was used in all procedures, and potentially contaminated material was handled in one NB2 safety laboratory using a Type 2 biological safety cabinet.

### Clinical samples collection

This study analyzed fecal samples from children under six years of age who presented symptoms of AGE, sent by the Central Laboratories of the states of Acre, Amazonas, Amapá, Pará, Rondônia, Roraima, and Tocantins located in the northern region of Brazil, by the Gastroenteritis Surveillance Network, in which the Evandro Chagas Institute, together with FIOCRUZ/RJ and Adolfo Lutz Institute/SP, are the three national reference laboratories for receiving and testing this material. Samples were collected over five years between January 2017 and December 2021.

### Suspension of fecal material

Fecal suspensions were prepared according to the World Health Organization (WHO) Polio Manual [23]. A mixture of 9 mL of phosphate buffered solution containing antibiotics (penicillin and streptomycin), 1 mL of chloroform, 1 g of glass beads, and 2 g of feces was prepared. Two aliquots of the supernatant were separated: one for inoculation into cell cultures and one for nucleic acid extraction. However, in some cases, the volume of feces available was very small, making it possible to obtain only one aliquot for RNA/DNA extraction.

### Cell cultures and viral isolation

The two cell lines used were HEp-2C (human laryngeal epidermoid carcinoma) and RD (human embryonic rhabidomyosarcoma) [23, 24].

Fecal suspensions were inoculated into the two cell lines after two days of growth. Microscopic observation was then performed for a period of five days (first passage). For the second passage, the tubes were subjected to three cycles of freezing in dry ice and thawing at 37°C in a water bath, aimed to rupture the cells and release possible existing viral particles. New inoculations (second passage) were performed with a period of five days of microscopic observation, totaling ten days to visualize the cytopathic effect (CPE).

### Nucleic acid extraction

Aliquots of the fecal suspension and cell fluids with CPE were subjected to DNA/RNA extraction using the QIAamp Viral RNA Mini kit (Qiagen, Hilden, Germany) on a QIAcube automated system (Qiagen), in accordance with the manufacturer’s instructions. Extracted nucleic acids were eluted in 60 μL of AVE elution buffer and immediately stored at -80°C until molecular analyses. For each extraction procedure, RNAse/DNAse-free water was used as a negative control. From these extracts, molecular techniques such as conventional PCR and RT-qPCR/qPCR were used to detect HAdV and EV, as well as to confirm the results obtained in cultures that showed CPE.

### Enterovirus and adenovirus detection by RT-qPCR and qPCR

EV detection was performed using the One-step GoTaq Probe 1-Step RT-qPCR System (Promega, Madison. WI, USA) by partial amplification of the 5′ NC region of the genome with previously published primers and probes [25, 26]. HAdV detection was performed by qPCR using previously described primers and probes [27] that amplify a region of the hexon gene. Reactions were performed using a QuantStudio 5 instrument (Applied Biosystems, Foster City, CA, USA). Samples with cycle threshold (CT) values of <40 were considered positive. For all the reactions, positive and negative controls were used to evaluate the efficacy of the reactions, reagents, and possible contamination.

### Conventional PCR detection and nucleotide sequencing

EV- and HAdV-positive samples obtained by RT-qPCR, qPCR, and/or cell cultures that showed CPE were subjected to conventional PCR, targeting a conserved region of the VP1 region of EV and the hexon gene of HAdV. Amplification of EV genetic material involves the synthesis of complementary DNA (cDNA) from extracted viral RNA using primers 222/224 [28, 29] and the primer pair Hex1-deg/Hex2-deg for HAdV amplification [30].

Reactions were carried out using Platinum Taq DNA Polymerase (Invitrogen, Carlsbad, CA, USA) following the manufacturer’s recommendations. The expected amplicon lengths for EV and HAdV were 762 and 301 base pairs (bp) respectively. The PCR fragments were purified using the QIAquick Gel Extraction or PCR Purification Kit (Qiagen), following the manufacturer’s recommendations. Purified amplicons were sequenced using a Big Dye Terminator v. 3.1 cycle sequencing kit (Applied Biosystems) on an ABI Prism 3730 DNA Analyzer (Applied Biosystems).

### Phylogenetic analysis

Nucleotide sequences were edited using BioEdit 7.2.5 software. Phylogenetic trees of the partial genes from the VP1 region (EV) and hexon (HAdV) were built in MEGA6 software [31] using the neighbor-joining and maximum likelihood methods, respectively, with the Jukes cantor model [32, 33]. We used 2000 replicates of bootstrap [34]. The partial nucleotide sequences from this study have been deposited in the GenBank database (http://www.ncbi.nlm.nih.gov) under the accession numbers OR735398–OR735429 and OR735352–OR735397.

### Statistical analysis

The data were analyzed by descriptive and analytical statistical tests using the Bio Estat 5.0 program [35], with a p-value ≤0.05 considered statistically significant. The agreement and replicability of the results between the ICC-PCR and qPCR/RT-qPCR techniques were obtained using the Screening Test and Kappa Index. A simple logistic regression test was used to verify the dependence of clinical variables (diarrhea, fever, and vomiting) and rotavirus vaccination status on the presence or absence of HAdV and NPEV. Chi-square and chi-square trend tests were used to evaluate the association between sex, age, and viral detection.

## Results

This study was carried out in states in the northern region of Brazil over five years (2017 to 2021) and involved fecal samples obtained by the Gastroenteritis Surveillance Network and collected from children with AGE under six years old. These specimens were analyzed for HAdV and NPEV.

A total of 801 fecal samples were analyzed using real-time PCR (RT-qPCR or qPCR), and 657 (82.0%) of these were subjected to viral isolation in HEp-2C and RD cell lineages, as they had sufficient fecal material for viral isolation.

The global viral positivity obtained by qPCR/RT-qPCR (Ct ranging from 12 to 38) was 47.6% (381/801), with 31.7% (254/801) corresponding to HAdV, 10.5% (84/801) to NPEV, and 5.4% (43/801) to co-detection. These viruses were detected throughout the study period, with the highest positivity observed in 2017 and 2021, with values of 54.1% (126/233) and 60.4% (81/134), respectively, and lower percentages in 2018 (36.5%, 46/126) and 2019 (39.0%, 78/200) (Table 1).

**Table 1:** Detection of human adenovirus (HAdV) and non-polio enterovirus (NPEV) by qPCR and RT-qPCR in 801 fecal samples from children with acute gastroenteritis, obtained in the northern region of Brazil, between 2017 and 2021.

Year	Samples % (Pos/Total)	HAdV % (Pos/Total)	NPEV % (Pos/Total)	Co-detection % (Pos/Total)	*X^2^* (*p*-valor)^a^
**2017**	54.1 (126/233)	34.8 (81/233)	11.6 (27/233)	7.7 (18/233)	33.733 (<0.0001)
**2018**	36.5 (46/126)	20.6 (26/126)	13.5 (17/126)	2.4 (03/126)	1.804 (0.1793)
**2019**	39.0 (78/200)	31.5 (63/200)	6.0 (12/200)	1,5 (03/200)	43.272 (<0.0001)
**2020**	46.3 (50/108)	18,5 (20/108)	17.6 (19/108)	10.2 (11/108)	0.032 (1.000)
**2021**	60.4 (81/134)	47.8 (64/134)	6.7 (9/134)	6.0 (8/134)	56.236 (<0.0001)
**Total**	47.6 (381/801)	31.7 (254/801)	10.5 (84/801)	5.4 (43/801)	

Table 2 lists the results obtained from 657 samples subjected to ICC-PCR (HEp-2C/RD cell lines) according to the year of sample collection and their comparison with the results obtained by qPCR. CPE was observed in 9.6% (63/657) of the inoculated samples, with positivity rates of 2.7% (18/657) for HAdV and 6.2% (41/657) for NPEV. Another four samples (0.6%) showed CPE, but the virus was not identified (NI) after molecular biology tests. The highest CPE identification rates were observed in 2017 (16.1%), with rates of 6.4% (10/155) for HAdV and 8.4% (13/155) for NPEV.

**Table 2:** Annual distribution of 657 fecal samples obtained from children with acute gastroenteritis tested by two methodologies: ICC-PCR (HEp-2c and RD cell lines) and qPCR/RT-qPCR for the presence of cytopathic effect (CPE) for human adenovirus (HAdV) and non-polio enterovirus (NEPV), from 2017 to 2021.

Year	N° of Inoculated samples*	HAdV Pos/total (%)			EVNP Pos/total (%)		
	Pos/total (%)	Inoc Cel	qPCR	+ECP/+qPCR	Inoc Cel	qPCR	+ECP/+qPCR
2017	25/155 (16,1)	10/155 (6,4)	63/155 (40,6)	10/63 (15,9)	13/155 (8,4)	30/155 (19,3)	13/30 (43,3)
2018	9/110 (8,2)	1/110 (0,9)	27/110 (24,5)	1/27 (3,7)	8/110 (7,3)	19/110 (17,3)	8/19 (42,1)
2019	10/186 (5,4)	1/186 (0,5)	66/186 (35,5)	1/66 (1,5)	8/186 (4,3)	15/186 (8,1)	8/15 (53,3)
2020	6/72 (8,3)	3/72 (4,2)	22/72 (30,5)	3/22 (13,6)	3/72 (4,2)	13/72 (18,0)	3/13 (23,1)
2021	13/134 (9,7)	3/134 (2,2)	72/134 (53,7)	3/72 (4,2)	9/134 (6,7)	17/134 (12,7)	9/17 (52,9)
TOTAL	63**/657 (9,6)	18/657 (2,7)	250/657 (38,0)	18/250 (7,2)	41/657 (6,2)	94/657 (14,3)	41/94 (43,6)

*Inoculated samples that showed cytopathic effects (CPE)

**Four samples showed CPE but were negative for the two viruses; HAdV, human adenovirus; EVNP, non-polio enterovirus.

The agreement between the results of both methodologies ranged from 64.4% for HAdV to 93.5% for NPEV, and the test replicability was poor for HAdV (kappa = 0.0798; p<0.0001) and good for NEPV (kappa = 0.6351; p<0.0001).

**Fig 1:**
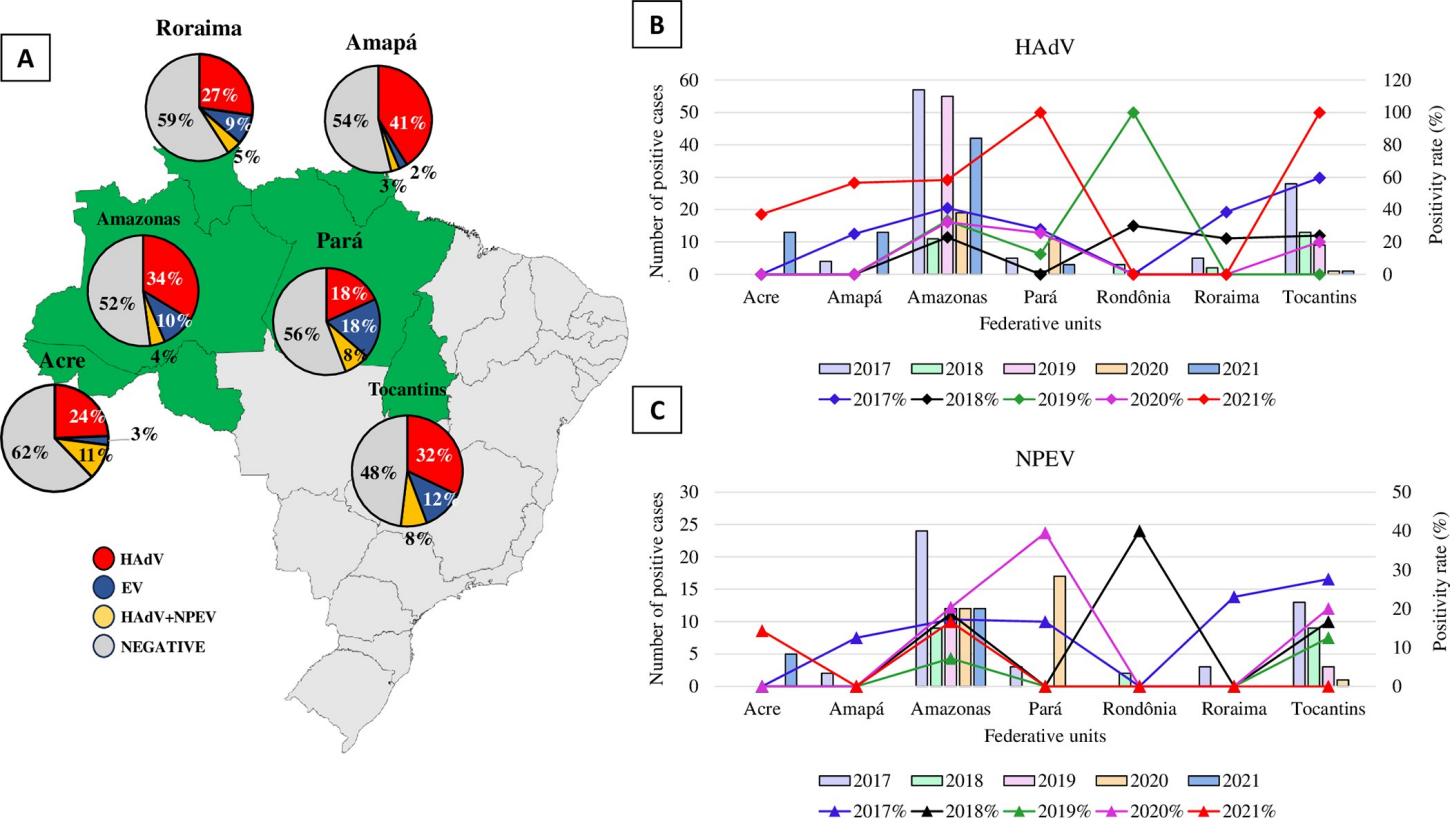
Detection of human adenovirus (HAdV) and non-polio enterovirus (NPEV) detected by qPCR and RT-qPCR in 801 fecal samples from children with acute gastroenteritis, in the northern region of Brazil between 2017 and 2021. Geographic distribution of positive cases for HAdV, NPEV and co-detection (HAdV+NPEV) in the seven Brazilian federal units. (A) Annual distribution by Brazilian federative unit of positive cases for (B) HAdV and (C) NPEV. Source: The map contains information from OpenStreetMap and OpenStreetMap Foundation, which is made available under the Open Database License. ©OpenStreetMap contributors.

Regarding the annual distribution of samples across the Brazilian federal units evaluated, these viruses were detected throughout the study period and Amazonas and Tocantins had the highest number of samples tested (484 and 131, respectively), both in inoculated cell cultures (HEp-2c and RD) and in qPCR for both viruses. The positivity in each federative unit ranged from 41% (Amapá) to 18% (Pará), whereas that for NEPV ranged from 18% (Pará and Rondônia) to 3% (Acre). The co-detection of both viruses occurred at lower and higher frequencies in Amapá (3%) and Acre (18%), respectively. Amazonas has accumulated the highest number of positive cases for both HAdV (N = 184/484, 38.0%) and NEPV (N = 69/484, 14.3%) over the years (Fig 1A–1C, S1 and S2 Files).

There was a difference between the positivity for HAdV and NPEV in males (p<0.0001; X^2^ = 52.545) and females (p<0.0001; X^2^ = 38.335); however, males were the most prevalent, with percentages of 40.3% and 17.6% for HAdV and NPEV, respectively (Table 3).

**Table 3:** Frequency of positive cases of human adenovirus (HAdV) and non-polio enterovirus (NPEV) detected by qPCR/RT-qPCR in relation to sex and age group in 801 fecal samples obtained from children with acute gastroenteritis in northern Brazil between 2017 and 2021.

Sex	HADV			NPEV		
	Male	Female	Total	Male	Female	Total
Age (months)	% (Pos/Total)	% (Pos/Total)	% (Pos/Total)	% (Pos/Total)	% (Pos/Total)	% (Pos/Total)
≤ 6	35.1 (19/54)	16.3 (8/49)	26.2 (27/103)	12.9 (7/54)	4.0 (2/49)	8.7 (9/103)
>6–12	40.7 (55/135)	34.2 (37/108)	37.8 (92/243)	13.3 (18/135)	13.8 (15/108)	13.5 (33/243)
>12–24	44.3 (47/106)	33.3 (34/102)	38.9 (81/208)	25.4 (27/106)	9.8 (10/102)	17.7 (37/208)
>24–60	34.0 (31/91)	28.9 (24/83)	31.6 (55/174)	17.5 (16/91)	20.4 (17/83)	18.9 (33/174)
>60	50.0 (16/32)	70.3 (19/27)	59.3 (35/59)	18.7 (6/32)	22.2 (6/27)	20.3 (12/59)
NI	50.0 (4/8)	50.0 (3/6)	50.0 (7/14)	12.5 (1/8)	33.3 (2/6)	22.4 (3/14)
**Total**	**40.3 (172/426)**	**33.3 (125/375)**	**37.0 (297/801)**	**17.6 (75/426)**	**13.8 (52/375)**	**15.8 (127/801)**

*NI: Not informed.

In terms of rates, the least affected by HAdV (26.2%) and NPEV (8.7%) were children up to six months of age, whereas the most affected were those over five years of age. HAdV infections were influenced by age (p = 0.0004; X^2^ = 12.497), whereas those caused by NPEV were not (p = 0.4269; X^2^ = 0.631). However, there was a tendency towards an increase in HAdV (p = 0.0175) and NPEV (p = 0.0087) infections due to the increase in the age groups analyzed.

To verify the association between the clinical aspects and positive cases of HAdV and NPEV, all samples previously positive for RV (unpublished data) and NoV [36] were removed from the analyses. The detection or co-detection of HAdV and NPEV did not influence the presence of diarrhea, fever, or vomiting (Table 4), and the report of a previous rotavirus vaccination was independent of the detection of HAdV and NPEV. Regarding mixed infections, the majority of HAdV- and NPEV-positive samples were associated with NoV infections (HAdV+NoV = 46.6%; NPEV+NoV = 11.3%). At least three viruses were detected in 29.3 and 9.4% of the samples, respectively. No co-detection of these four viruses was observed (Table 4).

**Table 4:** Frequency of positive cases of human adenovirus (HAdV) and non-polio enterovirus (NPEV) detected by qPCR/RT-qPCR was associated with clinical aspects, rotavirus vaccination, and mixed infections in fecal samples obtained from children with gastroenteritis in the northern region of Brazil between 2017 and 2021.

Variables	HAdV % (Pos/Total)	NPEV % (Pos/Total)	HAdV+NPEV % (Pos/Total)
Diarrhea*	***p* = 0.4548**	***p* = 0.3265**	***p* = 0.3495**
Yes	39.2 (123/314)	20.1 (48/239)	13.2 (29/220)
No	50.0 (6/12)	0 (0/6)	25.0 (2/8)
NI	47.4 (9/19)	33.3 (5/15)	0 (0/10)
Fever*	***p* = 0.8013**	***p* = 0.2301**	***p* = 0.1734**
Yes	40.5 (64/158)	18.1 (26/144)	13.8 (15/109)
No	39.0 (48/123)	19.0 (15/79)	7.4 (6/81)
NI	40.6 (26/64)	32.4 (12/37)	20.8 (10/48)
Vomit*	***p* = 0.7696**	***p* = 0.8636**	***p* = 0.0663**
Yes	37.9 (72/190)	18.0 (26/144)	13.2 (18/136)
No	39.6 (42/106)	19.0 (15/79)	4.5 (3/67)
NI	49.0 (24/49)	32.4 (12/37)	28.6 (10/35)
**RV vaccination**	***p* = 0.1360**	***p* = 0.3657**	***p* = 0.8060**
Yes	33.7 (55/163)	16.9 (22/130)	6.9 (8/116)
No	46.3 (19/41)	24.1 (7/29)	0 (0/22)
NI	45.4 (64/141)	23.8 (24/101)	23 (23/100)
**Mixed infections**			
RV positive	28.5 (37/130)	10.6 (11/104)	3.4 (5/146)
NoV positive	46.6 (62/133)	11.3 (9/80)	4.7 (7/149)
RV+NoV positive	29.3 (12/41)	9.4 (3/32)	0 (0/44)

NI: not informed.

*Cases positive for rotavirus and norovirus were not included in the analysis. P-values were obtained using a simple logistic regression test.

The seasonal distribution of HAdV and NPEV infections from January 2017 to December 2021 revealed that HAdV infections occurred throughout the years, except for a few months (August 2018, April 2019, and December 2020). NPEV was absent for several months during the study period, primarily in 2018 and 2019. Notably, there were months in which no samples were collected (June 2018, February and December 2019, April–August 2020, and January–March and August 2021). A predominance of HAdVs was observed in 2017. In March 2019, we received the highest number of samples (81) with 35.8 and 2.5% positivity rates for HAdV and NPEV, respectively.

**Fig 2:**
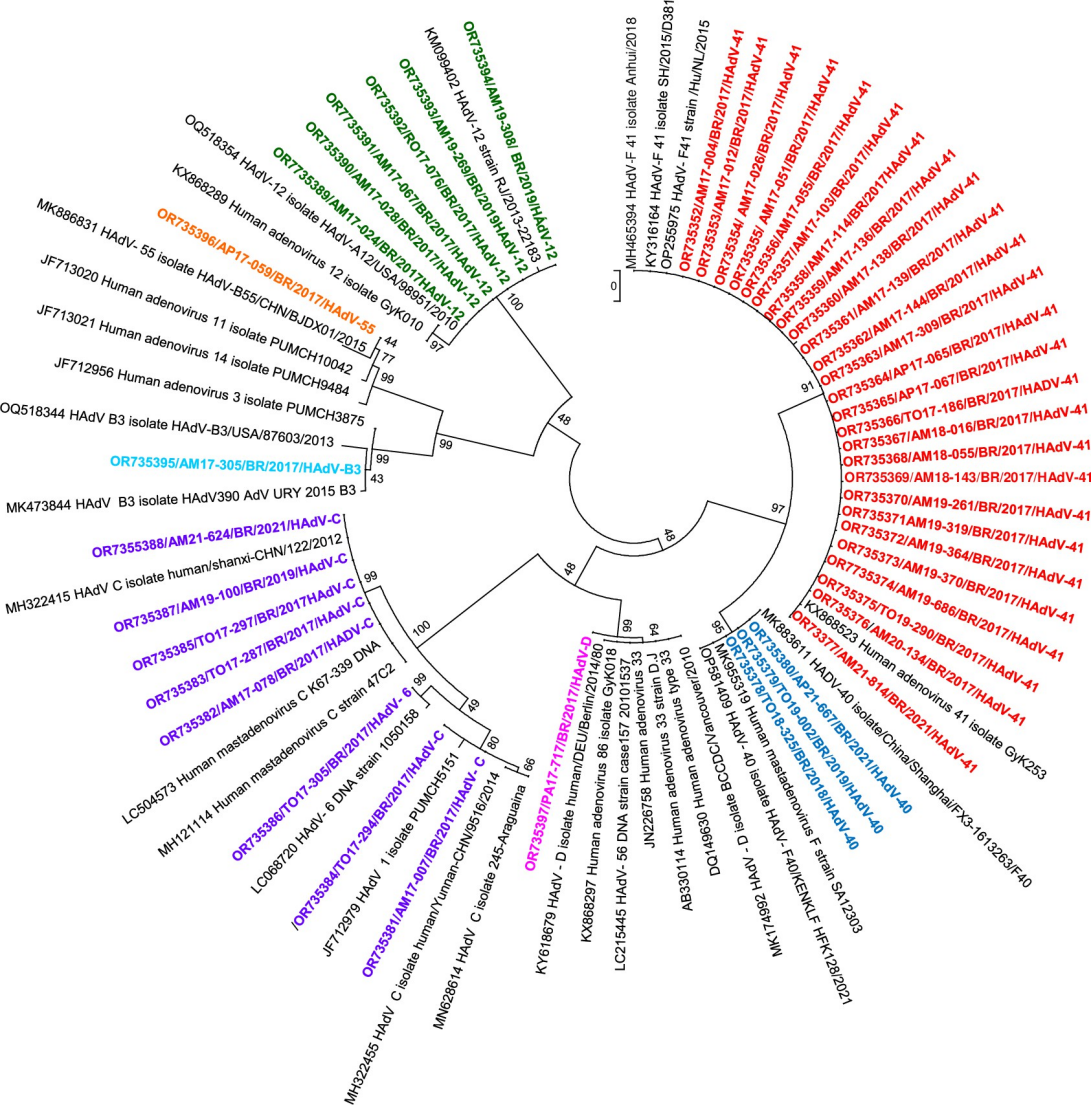
Phylogenetic analysis was based on a conserved nucleotide (nt) region of the hexon gene for adenovirus, detected in samples from children with acute gastroenteritis in the northern region of Brazil between 2017 and 2021. *Tree was inferred using maximum likelihood method based on Jukes-Cantor model. Genotypes are highlighted in red (HAdV-F41), dark blue (HAdV-F40), pink (HAdV-D), purple (HAdV-C), light blue (HAdV-B3), orange (HAdV-B55), and green (HAdV-A12). Reference strains were obtained from GenBank. A phylogenetic tree was constructed using MEGA 6 software with 2000 bootstrap replicates.

Of the 254 samples that tested positive by qPCR for HAdV only, 39.4% (100/254) were selected for nucleotide sequencing, with Ct values ranging between 17 and 38. In 46% (46/100) of the samples it was possible to perform viral taxonomic classification after sequencing. Of these, five species were identified (A, B, C, D, and F) and their genotypes are listed in Table 5. Species F, C, and A had the highest prevalence at 63% (n = 29), 17.4% (n = 8), and 13% (n = 6), respectively, whereas the least detected species were B (4.4%, n = 2) and D (2.2% n = 1). HAdV-F41 (56.5%) was the most prevalent genotype and was detected in Amazonas, Amapá, and Tocantins in 2017, and one case in 2021 in Amazonas (Fig 2).

**Table 5:** Human Adenovirus (HAdV) genotypes were detected in 46 fecal samples from children with acute gastroenteritis in northern Brazil between 2017 and 2021.

Species	Genotypes	N (%)	Year collection- BFU (N)
**A**	HAdV-A12	06 (13%)	2017-AM (3); RO (1)/ 2018-TO (1)/ 2019-AM (1)
**B**	HAdV-B3	01 (2.2%)	2017- AM (1)
	HAdV-B55	01 (2.2%)	2017- AP (1)
**C**	HAdV-C	07 (15.2%)	2017-AM (2); TO (3)/ 2019- AM (1)/ 2021- AM (1)
	HAdV-C6	01 (2.2%)	2017- TO (1)
**D**	HAdV- D	01 (2.2%)	2017- PA (1)
**F**	HAdV-F40	03 (6.5%)	2018- TO (1)/ 2019- TO (1)/ 2021- AP (1)
	HAdV-F41	26 (56.5%)	2017- AM (21); AP (2); TO (2)/ 2021- AM (1)

N: number of samples; BFU: Brazilian Federal unit; AM: Amazonas; RO: Rondônia; TO: Tocantins; AP: Amapá; PA: Pará.

**Fig 3:**
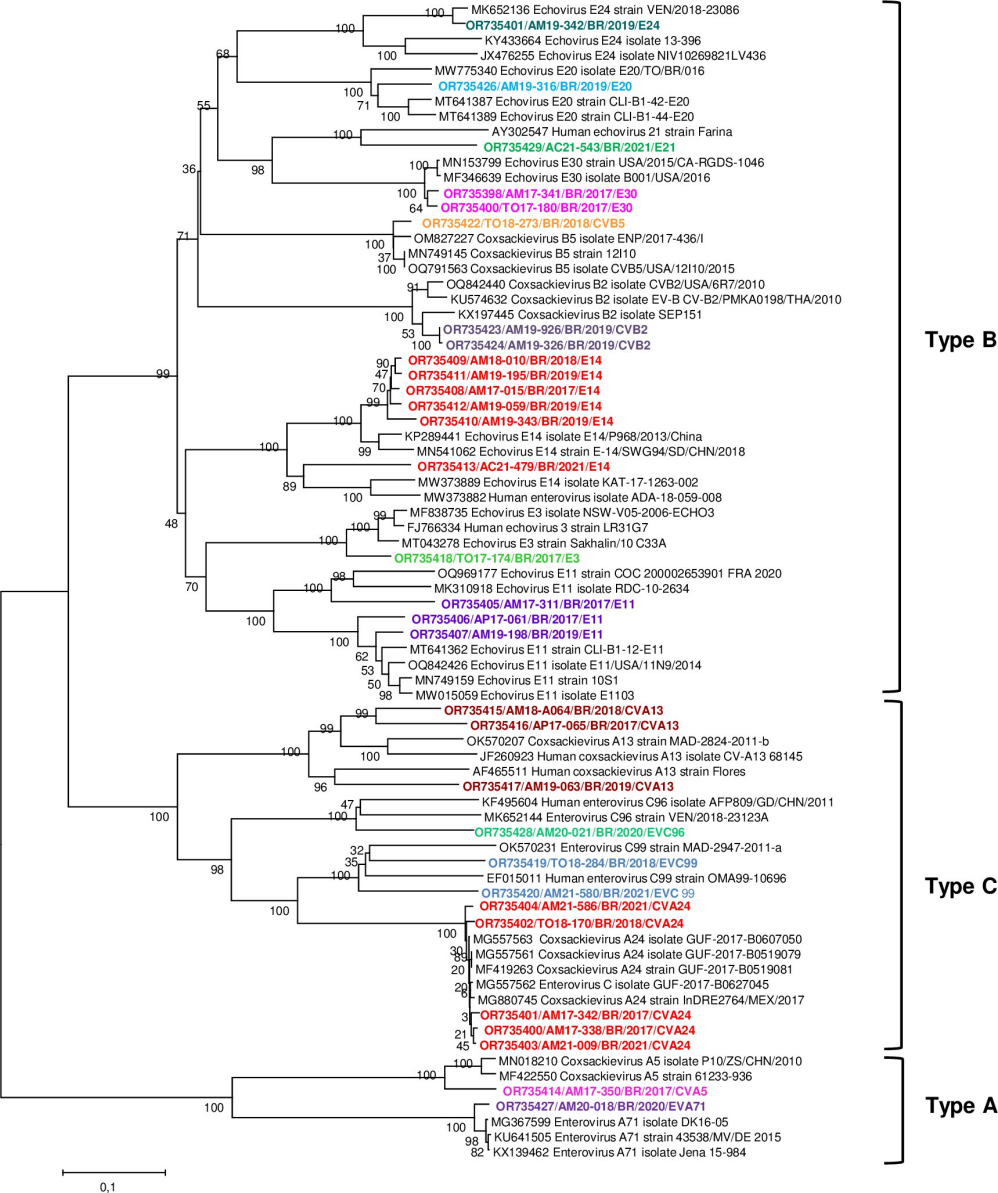
Phylogenetic analysis was based on a partial sequence of VP1 region of enteroviruses detected in samples from children with acute gastroenteritis in northern Brazil between 2017 and 2021. *The tree was inferred using the neighbor-joining method based on the Jukes-Cantor model. Reference strains were obtained from GenBank. A phylogenetic tree was constructed using the MEGA 6 software with 2000 bootstrap replicates.

For NPEV, of the 84 RT-qPCR-positive samples only for this virus, 44.0% (37/84) were selected for nucleotide sequencing based on the VP1 gene. Of these, 83.8% (31/37) could be classified, with three distinct species identified (A, B, and C) and 15 different types (Table 6). Species B was responsible for 60% (9/15) of the types detected (Fig 3).

**Table 6:** Non-polio enterovirus (NPEV) serotypes were detected in 31 fecal samples from children with acute gastroenteritis in northern Brazil between 2017 and 2021.

Species	Types	N (%)	Year collection- BFU (N)
**A**	CVA5	01 (3.2%)	2017- AM (1)
	EV-A71	01 (3.2%)	2020- AM (1)
**B**	E3	01 (3.2%)	2017- TO (1)
	E11	03 (9.7%)	2017- AM (1); AP (1)/ 2019- AM (1)
	E14	06 (19.3%)	2017- AM (1)/ 2018- AM (1)/ 2019- AM (3)/ 2021- AC (1)
	E20	01 (3.2%)	2019- AM (1)
	E21	01 (3.2%)	2021- AC (1)
	E24	01 (3.2%)	2019- AM (1)
	E30	02 (6.4%)	2017- AM (1); TO (1)
	CVB2	02 (6.4%)	2019- AM (2)
	CVB5	01 (3.2%)	2018- TO (1)
**C**	CVA13	03 (9.7%)	2017- AP (1)/ 2018- AM (1)/ 2019- AM (1)
	CVA24	05 (16.1%)	2017- AM (2)/ 2018- TO (1)/ 2021- AM (2)
	EV-C96	01 (3.2%)	2020- AM (1)
	EV-C99	02 (6.4%)	2018- TO (1)/ 2021- AM (1)

N: number of samples; BFU: Brazilian Federal unit; AM: Amazonas; TO: Tocantins; AP: Amapá; AC: Acre

## Discussion

This study was conducted over a five-year period using fecal samples obtained from the Gastroenteritis Surveillance Network previously tested for RV and NoV, providing information on the frequency of HAdV and NPEV in episodes of AGE. Important epidemiological, clinical, and molecular data are presented, considering the simultaneous evaluation of these viruses to provide information on the specific characteristics of viral etiology, especially because of the few studies related to HAdV and NPEV in the Amazon region.

In Brazil, the circulation of HAdVs in gastroenteritis cases has been reported in other studies. In a previous study carried out in the southeast, south, and northeast regions of Brazil, the detection rates of HAdV in cases of gastroenteritis were 21.2, 22.5, and 31.9%, respectively [19], which is similar to the rate of 37% found in the current study in the northern region of Brazil but higher than the frequency of HAdV demonstrated in another retrospective study carried out in the central-west regions and in parts of the southeast and south regions (3.9%) [37].

The north and northeast regions are the poorest in the country [38], presenting precarious scenarios such as lack of hygiene and open sewage, which may contribute to the spread of various etiological agents, including the viruses investigated in the present study.

In the northern region of Brazil, another study conducted in Belém demonstrated a detection rate of 26.3% (46/175) for EV using conventional PCR techniques [21]. Another survey in the same city observed 76.5% EVs in children with gastroenteritis [39]. Previous studies have reported EV as being the second or third most common viral agents in gastrointestinal infections, similar to what was observed in the present study [40–42].

Research has demonstrated the circulation of HAdV and EVNP throughout the Brazilian territory; however, in the current study, Amazonas presented the highest percentage of positivity, largely due to better epidemiological surveillance in this location, which culminated in the largest number of sample collection clinics, when compared to other locations in Brazil. This reinforces the need to intensify the surveillance of diarrheal syndrome, especially in remote locations where there is still no epidemiological information on the agents that cause this important issue in pediatric health.

Regarding age group, higher frequencies of viral infection were observed in the 12–24 month (38.9%) and 24–60 month (18.9%) groups for AdV and EV, respectively. Previous studies have demonstrated that for both viruses, the age group under five years is the most affected worldwide [19, 43–45]. Interestingly, the statistical inference applied to the data in the present study suggests a growing trend towards an increase in the number of HAdV (p = 0.0175) and EV (p = 0.0087) infections according to the progression of the age group of patients with AGE. This corroborates that older children may be more susceptible to infection by both viruses; however, larger epidemiological studies are required to better understand the factors involved in infection in relation to patient age.

Regarding the most affected sex, 40% of the patients were male, and 33.3% were females. Other studies worldwide have revealed different predominance, such as the one that occurred in Saudi Arabia between 2017 and 2018, which showed a predominance of females (10.4%) over males (3.8%) [44]. In Thailand, research carried out over 7 years found that viral infections between male and female groups were very similar, with rates of 6.6 and 7.7%, respectively [46].

It is possible that the immune systems of males and females respond differently to viral infections. More robust research on the reasons why one sex may be more affected by these agents than the other is necessary to infer any cause-and-effect relationship. However, the present study observed through statistical inference that HAdV infection in different sexes may be substantially greater than EV infection for reasons that are still poorly understood and require further investigation.

The current study demonstrated a 31.7% co-detection rate between both viruses. Martinez et al. [47] also found cases of co-detection at a high percentage (48%), which was higher than that of other viruses they investigated. The data from the present study shed light on the high frequency of HAdV and NPEV in cases of childhood diarrhea, although it was not possible to determine which of them was associated with the patients’ clinical manifestations, such as diarrhea.

The presence of HAdV and NPEV in the analyzed samples was confirmed using qPCR and ICC-PCR. Standardized cell cultures for EV explain the success of their isolation compared to that of HAdVs. There are other more specific cell lines for HAdV isolation, mainly HAdV-F40 and-F41, but these were not available in our laboratory. Therefore, by comparing the two methods, the molecular techniques are more useful and sensitive for the detection of both viruses.

Previous research carried out in Belém showed a detection rate of 26.3% (46/175) for EV using conventional PCR techniques, with an agreement of 78.3% (36/46) when compared to inoculation in Hep-2 and RD cell cultures [21], which were much higher than those observed in the present study (15.9 and 43.6%, respectively). Another investigation conducted in Belém between 1982 and 2019 [39] also showed a higher positivity rate of 50.8% (119/234), using the same technique (RT-qPCR).

Most EVs are cytopathogenic, and their diagnosis is recommended by the WHO through viral isolation, as most of them are cultivable [23]. Although very sensitive and advantageous for obtaining the viral agent, diagnosis using this technique may take longer than RT-qPCR, but with greater sensitivity and specificity for identifying EVs. Regarding HAdV, there has been less detection by cell culture, most likely because it is difficult and slow to grow, and diagnosis by PCR and qPCR is recommended.

Based on the phylogenetic analyses performed in this study, five HAdV species were identified. Species F (types 40 and 41), recognized as a prominent cause of diarrhea and diarrhea-associated mortality in young children worldwide, was the most prevalent, accounting for 63% of the genotyped samples, with HAdV-F41 present in 56.5% of the samples and HAdV-40 in 6.5%. This finding corroborates with other studies already carried out in Brazil that demonstrate the involvement of these genotypes in cases of gastroenteritis [19, 20, 48].

Genotype A-12, which is also related to gastroenteric episodes, was detected in 13% of the samples. Furthermore, species B, C, and D, which are related to other diseases, were also found in low percentages, with the exception of C, which was the second most detected, with a positivity rate of 15.2%; however, the impact of this detection on children with gastroenteritis is still unclear. Studies have shown that after primary infection, HAdV-C DNA can persist in lymphoid cells in a latent form, and that asymptomatic and intermittent excretion of this virus through feces can be observed for many years [49, 50].

Although we identified the presence of diarrhea, vomiting and fever in EV-positive samples but negative for RV and NoV, it is not possible to affirm that these viruses caused the symptoms, as serological tests would be necessary to confirm the increase in antibody titers and proof of being the same type of EV isolated in feces [51], which was not done and was a limitation of this study.

Notably, NEPV was detected in fecal samples from children with AGE; however, no information was provided on other clinical conditions. Therefore, some of the coxsackieviruses detected in the feces have been associated with the following illnesses: hand-foot-and-mouth disease (CVA5, CVB5), acute flaccid paralysis (CVA13), outbreaks of hemorrhagic conjunctivitis (CVA24), meningoencephalitis (CVB2), encephalitis, aseptic meningitis, paralysis, and herpangina (CVB5) [52–56].

EV-A71 is related to outbreaks of uncomplicated hand-foot-and-mouth disease, herpes or febrile illness, and central nervous system involvement, such as aseptic meningitis, myoclonic tremor, polio-like syndrome, encephalitis, encephalomyelitis, and cardiopulmonary failure due to severe rhombencephalitis [57, 58].

EV-C96 and C99 are more recently discovered types isolated from patients with acute flaccid paralysis, hand-foot-and-mouth disease, diarrhea, healthy individuals, and environmental samples. C99 was first reported in South America in 2019 and in a fecal sample collected in 2013 in Tocantins State, North Brazil [22, 59, 60].

E11 and E30 are the most detected echoviruses. E11 is associated with neonatal diseases, including fulminant hepatitis, and E30 is associated with outbreaks and sporadic cases of meningitis in Brazil and other European countries [17, 61, 62]. Other detected echoviruses are rarely reported and have low prevalence [63].

## Conclusions

This study demonstrated the prevalence, clinical data, seasonal distribution, and diversity of genotypes and serotypes of HAdV and NPEV circulating in children with AGE in the Amazon region of northern Brazil.

A five-year surveillance study was conducted, providing information on the circulation and genetic diversity of HAdV and NPEV associated with cases of AGE. It also revealed a high incidence of HAdV-F41 in cases of diarrhea, a fact already described in the literature that corroborates our findings.

This reinforces the need to adapt current surveillance strategies to monitor the emergence and reemergence of NPEV in a timely manner. These results suggest that the circulation of these pathogens in the Amazon region has been increasing, owing to factors such as basic sanitation, hygiene, and the agglomeration of closed groups. These results suggested that EV should be included in the diagnosis of AGE.

## Supporting information

S1 File Annual distribution by Brazilian federal unit of HAdV-positive cases from fecal samples obtained of acute gastroenteritis in children, detected by viral isolation in HEp -2c and RD cell lines and qPCR, from 2017 to 2021. (DOC)

S2 File Annual distribution by Brazilian federative unit of non poliovirus enterovirus (NPEV) positive cases from fecal samples obtained of acute gastroenteritis in children, detected by viral isolation in HEp -2c and RD cell lines and RT-qPCR in the period from 2017 to 2021. (DOC)
