# Supplementary material for: Epidemiology and molecular detection of human adenovirus and non-polio enterovirus in fecal samples of children with acute gastroenteritis: A five-year surveillance in northern Brazil
Source: PLoS One. 2024 Aug 2;19(8):e0296568. doi: 10.1371/journal.pone.0296568 (PMC11296658; doi:10.1371/journal.pone.0296568)
Supplement: S1 File — (DOC) [file pone.0296568.s001.doc]

Supplementary material 1. Annual distribution by Brazilian federal unit of HAdV-positive cases from fecal samples obtained of acute gastroenteritis in children, detected by viral isolation in HEp -2c and RD cell lines and qPCR, from 2017 to 2021.

| **Cytopathic Effect + HAdV positive PCR/ Total of Samples Inoculated (% HAdV positivity)** | | | | | | | | |
| --- | --- | --- | --- | --- | --- | --- | --- | --- |
| **Year** | **Acre** | **Amazonas** | **Amapá** | **Pará** | **Rondônia** | **Roraima** | **Tocantins** | **Total^b^** |
| **2017** | **-**^a^ | 3/85 (3.5) | 1/15 (6.7) | 1/13 (7.7) | - | 0/3 | 5/39 (12.8) | 10/155 (6.4) |
| **2018** | - | 1/45 (2.2) | - | 0/2 | 0/7 | 0/3 | 0/53 | 1/110 (0.9) |
| **2019** | - | 1/160 (0.6) | - | 0/1 | 0/1 | - | 0/24 | 1/186 (0.5) |
| **2020** | 0/1 | 2/59 (3.4) | - | 0/7 | - | - | 1/5 (20.0) | 3/72 (4.2) |
| **2021** | 0/35 | 3/72 (4.2) | 0/23 | 0/3 | - | - | 0/1 | 3/134 (2.2) |
| **Total** | 0/36 | 10/421 (2.4) | 1/38 (2.6) | 1/26 (3.8) | 0/8 | 0/6 | 6/122 (4.9) | 18/657 (2.7) |
| **HAdV positive by qPCR/ Total of Samples Tested (% HAdV positivity)** | | | | | | | | |
| **2017** | - | 57/139 (41.0) | 4/16 (25.0) | 5/18 (27.8) | - | 5/13 (38.5) | 28/47 (59.6) | 99/233 (42.5) |
| **2018** | - | 11/48 (22.9) | - | 0/5 | 3/10 (30.0) | 2/9 (22.2) | 13/54 (24.1) | 29/126 (23.0) |
| **2019** | 0/1 | 55/166 (33.1) | - | 1/8 (12.5) | 1/1 (100) | - | 9/24 (37.5) | 66/201 (32.8) |
| **2020** | 0/1 | 19/59 (32.2) | - | 11/43 (25.6) | - | - | 1/5 (20.0) | 31/108 (28.7) |
| **2021** | 13/35 (31.4) | 42/72 (58.3) | 13/23 (56.5) | 3/3 (100) | - | - | 1/1 (100.0) | 72/134 (53.7) |
| **Total** | 13/37 (35.1) | 184/484 (38.0) | 17/39 (43.6) | 20/77 (26.0) | 4/11 (36.4) | 7/22 (31.8) | 52/131 (39.7) | 297/801 (37.2) |

^a^No samples collected.

^b^Co-detection among HAdV and NPEV were included.
