# Supplementary material for: Epidemiology and molecular detection of human adenovirus and non-polio enterovirus in fecal samples of children with acute gastroenteritis: A five-year surveillance in northern Brazil
Source: PLoS One. 2024 Aug 2;19(8):e0296568. doi: 10.1371/journal.pone.0296568 (PMC11296658; doi:10.1371/journal.pone.0296568)
Supplement: S2 File — (DOC) [file pone.0296568.s002.doc]

Supplementary material 2. Annual distribution by Brazilian federative unit of non poliovirus enterovirus (NPEV) positive cases from fecal samples obtained of acute gastroenteritis in children, detected by viral isolation in HEp -2c and RD cell lines and RT-qPCR in the period from 2017 to 2021.

| **Cytopathic Effect + NPEV positive PCR/Total of Samples Inoculated (% NPEV positivity)** | | | | | | | | |
| --- | --- | --- | --- | --- | --- | --- | --- | --- |
| **Year** | **Acre** | **Amazonas** | **Amapá** | **Pará** | **Rondônia** | **Roraima** | **Tocantins** | **Total^b^** |
| **2017** | **-**^a^ | 10/85 (11.7) | 1/15 (6.7) | 0/13 | **-** | 0/3 | 2/39 (5.1) | 13/155 (8.3) |
| **2018** | - | 3/45 (6.7) | - | 0/2 | 0/7 | 0/3 | 5/53 (9.4) | 8/110 (7.3) |
| **2019** | - | 7/160 (4.4) | - | 0/1 | 0/1 | **-** | 1/24 (4.2) | 8/186 (4.3) |
| **2020** | 0/1 | 3/59 (5.1) | - | 0/7 | **-** | **-** | 0/5 | 3/72 (4.2) |
| **2021** | 3/35 (8.6) | 6/72 (8.3) | 0/23 | 0/3 | **-** | **-** | 0/1 | 9/134 (6.7) |
| **Total** | 3/36 (8.3) | 29/421 (6.9) | 1/38 (2.6) | 0/26 | 0/8 | 0/6 | 8/122 (6.6) | 41/657 (6.2) |
| **NPEV positive by RT-qPCR/ Total of Samples Tested (% NPEV positivity)** | | | | | | | | |
| **2017** | **-** | 24/139 (17.3) | 2/16 (12.5) | 3/18 (16.7) | **-** | 3/13 (23.1) | 13/47 (27.7) | 45/233 (19.3) |
| **2018** | - | 9/48 (18.7) | **-** | 0/5 | 2/10 (20.0) | 0/9 | 9/54 (16.7) | 20/126 (15.9) |
| **2019** | 0/1 | 12/166 (7.2) | **-** | 0/8 | 0/1 | **-** | 3/24 (12.5) | 15/200 (7.5) |
| **2020** | 0/1 | 12/59 (20.3) | **-** | 17/43 (39.5) | **-** | **-** | 1/5 (20.0) | 30/108 (27.8) |
| **2021** | 5/35 (8.6) | 12/72 (16.7) | 0/23 | 0/3 | **-** | **-** | 0/1 | 17/134 (12.7) |
| **Total** | 5/37(13.5) | 69/484 (14.3) | 2/39 (5.1) | 20/77 (26.0) | 2/11 (18.2) | 3/22 (13.6) | 25/131 (19.1) | 127/801 (15.8) |

^a^No samples collected.

^b^Co-detection among HAdV and NPEV were included
